# Anthropogenic Pressure on Tree Species Diversity, Composition, and Growth of *Balanites aegyptiaca* in Dinder Biosphere Reserve, Sudan

**DOI:** 10.3390/plants10030483

**Published:** 2021-03-04

**Authors:** Elmugheira M. I. Mohammed, Elhag A. M. H., Patrick A. Ndakidemi, Anna C. Treydte

**Affiliations:** 1 Department of Sustainable Agriculture, Biodiversity Conservation and Ecosystem Management, School of Life Sciences and Bioengineering, The Nelson Mandela African Institution of Science and Technology, Arusha P.O. Box 447, Tanzania; patrick.ndakidemi@nm-aist.ac.tz (P.A.N.); anna.treydte@nm-aist.ac.tz (A.C.T.); 2 Department of Forest Management Science, Faculty of Forest Science and Technology, University of Gezira, Wad Medani P.O. Box 20, Sudan; 3 Department of Basic Science, College of Natural Resources and Environmental Studies, University of Bahri, Ministry of Higher Education and Scientific Research, Khartoum North (Al-Kadaro District), Khartoum P.O. Box 1660, Sudan; remotesensing9@gmail.com; 4 Ecology of Tropical Agricultural Systems, Hans-Ruthenberg Institute, University of Hohenheim, 70593 Stuttgart, Germany

**Keywords:** Balanitaceae, conservation, forests, rangelands, species dynamics, sustainable management

## Abstract

Anthropogenic disturbances, such as illegal harvesting and livestock browsing, often affect natural forests. However, the resulting tree species diversity, composition, and population structure have rarely been quantified. We assessed tree species diversity and importance value indices and, in particular, *Balanites aegyptiaca* (L.) Del. population structure, across 100 sample plots of 25 m × 40 m in disturbed and non-disturbed sites at the Dinder Biosphere Reserve, Sudan, from April 2019 to April 2020. We found that the tree species diversity in non-disturbed sites was more than double that of disturbed sites (*p* < 0.001, *T* = 32.6), and seedlings and saplings comprised more than 72% of the entire tree population (*F_2,48_* = 116.4, *p* = 0.034; *F_2,48_* = 163.2, *p* = 0.021, respectively). The tree density of *B. aegyptiaca* in the disturbed site was less than half that of the non-disturbed site (*p* = 0.018, *T* = 2.6). *Balanites aegyptiaca* was seven times more aggregated in disturbed sites compared to more regularly spaced trees in non-disturbed sites (*T* = 39.3 and *p* < 0.001). The poor *B. aegyptiaca* population status of the disturbed site shows that the conservation of this vulnerable species is essential for a sustainable management and utilization scheme.

## 1. Introduction

Natural forests, forest plantations, biosphere reserves, and other protected areas have always consolidated human lives ecologically and economically [1,2,3]. The ecological support of the forests can be observed through its hydrological functions, carbon sequestration, sites of recreation, biodiversity protection and conservation [1,4,5,6]. Their economic contribution includes the provision of food, timber and non-timber products, fodder for livestock, medicinal extracts, and shelter for humans and animals [2,7,8]. Biosphere reserves constitute a massive stock of biodiversity and perform a principal role towards the satisfaction of various needs for the local communities worldwide [9,10]. Particularly in sub-Saharan countries and the Sahel region, the recent and rapidly increasing over-exploitation and other human disturbances may strongly degrade the dynamics of these resources [11,12].

In eastern Africa, trees and shrubs, often growing inside or neighboring protected areas, provide multiple products and services to local people [13,14]. This high anthropogenic pressure can disturb the population dynamics of trees by eliminating their natural regeneration and the removal of mature trees [11,15,16]. Various tree species in the Dinder Biosphere Reserve (DBR), the oldest protected area in Sudan which borders three states (Gedaref, Sinnar, and the Blue Nile), are facing continuous disturbances from local communities through overgrazing and browsing by livestock, illegal harvesting, the debarking of tree stems and removal of branches [17,18].

While browsing likely affects the seedlings and saplings of highly palatable tree species [19,20,21], the human impact focuses on mature tree resources such as stem bark for medicine [22,23], stem and branches for timber [24,25] or firewood production [26,27,28,29]. Despite an ongoing high human and livestock pressure, information regarding the health status of the common tree species in the Dinder Biosphere Reserve is limited. Data about natural regeneration, the ratio of seedlings to saplings and mature trees, as well as the tree crown conditions and basal area contribution for these common trees are scarce.

Among the most frequently used tree species by local communities is *Balanites aegyptiaca* (L.) Del. This native and multipurpose tree species in Sudan is used for food, feed and medicine [30,31]. Its leaves are edible and its fruits are a source of income for local people around the protected areas and forest reserves [32,33,34]. Extracts from the roots, leaves, bark, and wood of the species have been used as an antioxidant, antifungal, and anticancer products [35,36,37,38]. The fruits of *B. aegyptiaca* are characterized by a high content of oil suitable for biodiesel production [39]. Despite these uses, little is known about this species’ population in protected and unprotected areas, and the possible effect of illegal harvesting and livestock grazing on the population structure of *B. aegyptiaca* stands. Therefore, the current study aims to analyze the influence of anthropogenic pressure, in particular illegal harvesting and livestock browsing, on the tree species diversity, composition, and growth of *B. aegyptiaca* in the Dinder Biosphere Reserve, Sudan.

Livestock grazing and browsing affect the natural regeneration of trees and reduce the species diversity in frequently grazed/browsed areas [19,40,41]. Results on diversity responses to disturbance are indefinite; diversity indices are often lower in disturbed systems, as only limited tree species survive intensive browsing and illegal felling [24,42]. On the other hand, diversity might be higher, as plant communities can be enriched by pioneer species using the newly opened up space [1,43]. Further, the population structure and dynamics of trees as reflected by size, basal area, and stage of development can show the stage of recovery/resilience of the system [26]. We hypothesized that disturbed sites in the DBR had lower tree species diversity than the non-disturbed sites, and that only a few tree species will dominate. We investigated in particular whether species with a high importance value index (IVI), an index that quantifies the ecological significance of a species in a specific community [43,44], will be negatively affected by human and livestock disturbance. We further hypothesized that natural regeneration and growth will be severely reduced in the disturbed sites, while larger crown width and higher tree densities will be expected for *B. aegyptiaca* in the non-disturbed site.

## 2. Results

### 2.1. Low Tree Species Diversity and Density in Disturbed Sites

The non-disturbed site had a tree species richness that was more than twice as high as that in the disturbed site (*p* < 0.001, *T* = 19). The diversity index was also more than double that of the disturbed site, with a similar trend for Simpson’s evenness index (*p* < 0.001, *T* = 32.6; and *p* < 0.05, *T* = 28.0, respectively).

**Figure 1:**
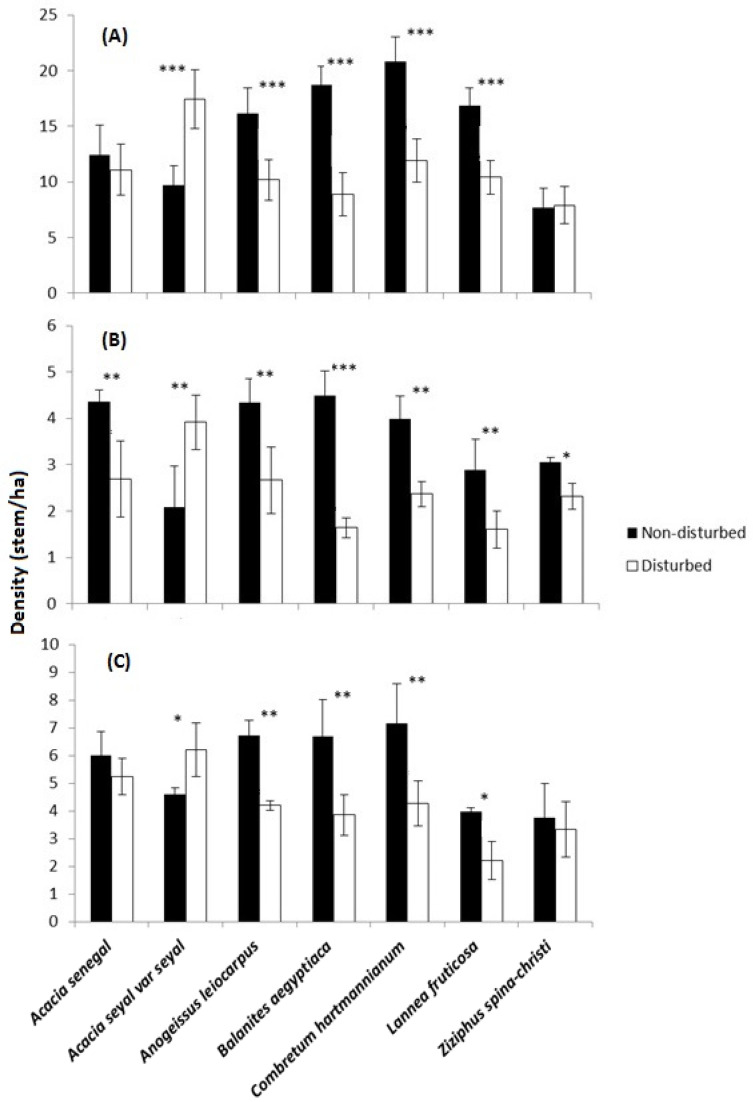
Average (±SE) density (stem/ha) of the seven most common tree species in the non-disturbed and disturbed sites of the Dinder Biosphere Reserve in the year of 2020. (**A**) Mature trees; (**B**) saplings; (**C**) seedlings. Asterisks above the bars show significant differences across the sites for each species according to Tukey’s Post-Hoc tests (* = *p* < 0.05; ** = *p* < 0.01; *** = *p* < 0.001; *n* = 408).

The density of the seven most common mature trees (*F_6,406_* = 20.3, *p* = 0.001), saplings (*F_6,406_* = 19.1, *p* = 0.011), and seedlings (*F_6,406_* = 17.3, *p* = 0.021) was significantly different between the non-disturbed and disturbed sites, with generally higher densities in the non-disturbed sites (Figure 1). This significant difference was mainly visible for *Combretum hartmannianum* Schweinf, *B. aegyptiaca, Anogeissus leiocarpus* (DC.) Guill and Perr., and *Lannea fruticosa* (Hochst.) Engl tree species, while *Acacia seyal* Del., showed a contrasting pattern, having higher densities in disturbed sites (Figure 1). The seedling density for both *C. hartmannianum* and *B. aegyptiaca* in the non-disturbed site was almost double that of the disturbed site (Figure 1).

**Figure 2:**
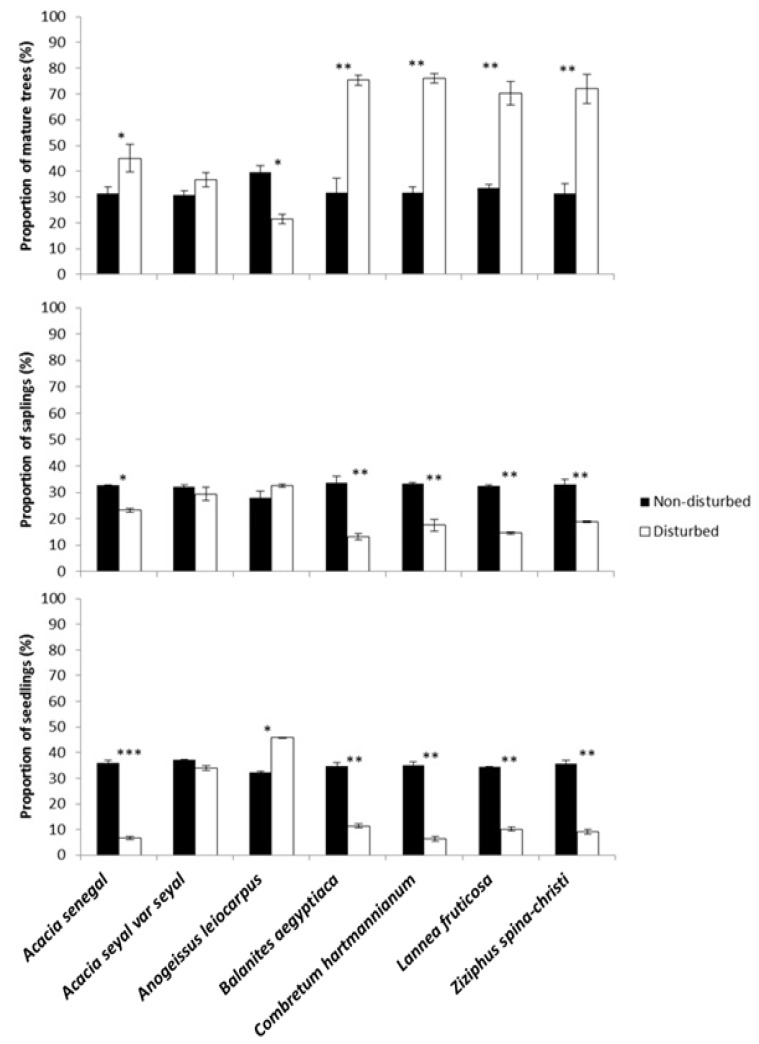
The proportions of mature trees, saplings and seedlings (%) of the seven most common tree species in the non-disturbed and disturbed sites of the Dinder Biosphere Reserve in the year of 2020. Asterisks above the bars show significant differences across the sites for each species according to Tukey’s Post-Hoc tests (* = *p* < 0.05; ** = *p* < 0.01; *** = *p* < 0.001; *n* = 406).

Moreover, our findings showed that the proportion of the seven most common tree species were significantly different for adult trees (*F_6,404_* = 22.3; *p* = 0.012), saplings (*F_6,404_* = 31.1; *p* = 0.01), and seedlings (*F_6,404_* = 27.6; *p* = 0.013) between the non-disturbed and disturbed sites, with high percentages of seedlings and saplings in the non-disturbed site and adult trees dominating the disturbed site (Figure 2). The percentage of the adult trees for *B. aegyptiaca, C. hartmannianum, L. fruticosa,* and *Ziziphus spina-christi* (L.) Desf., in the disturbed site were double that in non-disturbed site, while their seedlings were more than three times as high as those in the disturbed site (Figure 2). However, *Anogeissus leiocarpus* illustrated an inverse pattern, with higher proportions of seedlings and saplings in the disturbed sites and more adult trees in the non-disturbed sites.

### 2.2. High Importance Value Index (IVI) and High Dominance in Disturbed Sites

Both the disturbed and non-disturbed sites were dominated by *B. aegyptiaca, C. hartmannianum* and *Acacia seyal var seyal*, all of which had a species importance value index (IVI) of >28 (Table A1 and Table A2). *Balanites aegyptiaca* was the most dominant tree species in the non-disturbed site, but less so in the disturbed site (*F_1,43_* = 138.8, *p* < 0.001). The importance value index (IVI) for the frequently encountered tree species was significantly higher in the disturbed vs. non-disturbed sites (*F_6,406_* = 88.3, *p* = 0.0123).

We also found that relative abundance, dominance, and frequency for most tree species in the DBR were lower in the disturbed sites, except for *Acacia seyal* and *Acacia senegal* (L.) Willd., demonstrating the inverse trend (*F_1,43_* = 205.4, *p* < 0.001). *Acacia seyal* was four times as dominant in the disturbed than in the non-disturbed site, while its abundance was almost double that of the non-disturbed site (*F_1,43_* = 164.3, *p* < 0.01). The same trend was observed for the importance value index (Table A1, Table A2 and Table A3). Further, the total number of mature trees for *B. aegyptiaca, Anogeissus leiocarpus* and *Lannea fruticosa* in the non-disturbed site was almost double and three times that of the disturbed site, respectively (Table A3).

**Figure 3:**
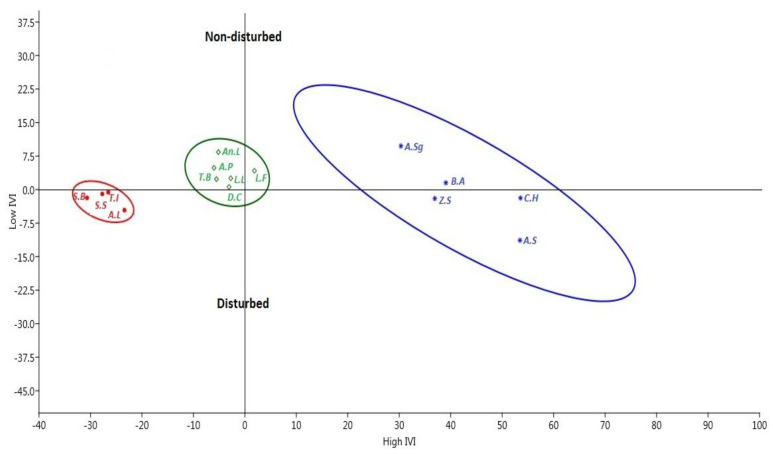
The projection of clustered groups of the common tree species at the disturbed and non-disturbed sites in the Dinder Biosphere Reserve, Sudan which was performed in the principal component analysis (PCA) based on the importance value index (IVI) values. Blue colored species are those with highest IVI, i.e., *A.S* = *Acacia seyal var seyal*, *C.H* = *Combretum hartmannianum*, *Z.S* = *Ziziphus spina-christi*, *B.A* = *Balanites aegyptiaca*, *A.Sg* = *Acacia senegal*. Green colored have intermediate IVI, i.e., *An.L* = *Anogeissus leiocarpus*, *A.P* = *Acacia polyacantha* Will, *L.F* = *Lannea fruticosa*, *D.C* = *Dichrostachys cinerea*, *T.B* = *Terminalia brownii*, while the red colored group has a low IVI, i.e., *T.I* = *Tamarindus indica*, *S.B = Sclerocarya birrea* (A. Rich) Hochst, *A.L = Acacia seyal (Del.) var fistula (Schweinf) Oliv*, and *S.S = Sterculia setigera*.

Our principal component analysis on the IVI values showed that both the highest and lowest IVI values were found in disturbed sites. The first group (tree species with high IVI) was dominated by *Acacia seyal, Combretum hartmannianum, Balanites aegyptiaca, Ziziphus spina-christi,* and *Acacia senegal*), while the second and third groups (medium and low IVI values, respectively) were discriminated by *Anogeissus leiocarpus, Acacia polyacantha, Terminalia brownii* Fresen, *Lannea fruticosa, Dichrostachys cinerea* (L.) Wight and Arn., *Tamarindus indica* L., and *Sterculia setigera* Del (Figure 3). In addition, the analysis effectively expressed and explained 78% of the variation among the disturbed and non-disturbed sites.

### 2.3. Natural Regeneration and Growth form Distribution Are Highly Limited in Disturbed Sites

**Figure 4:**
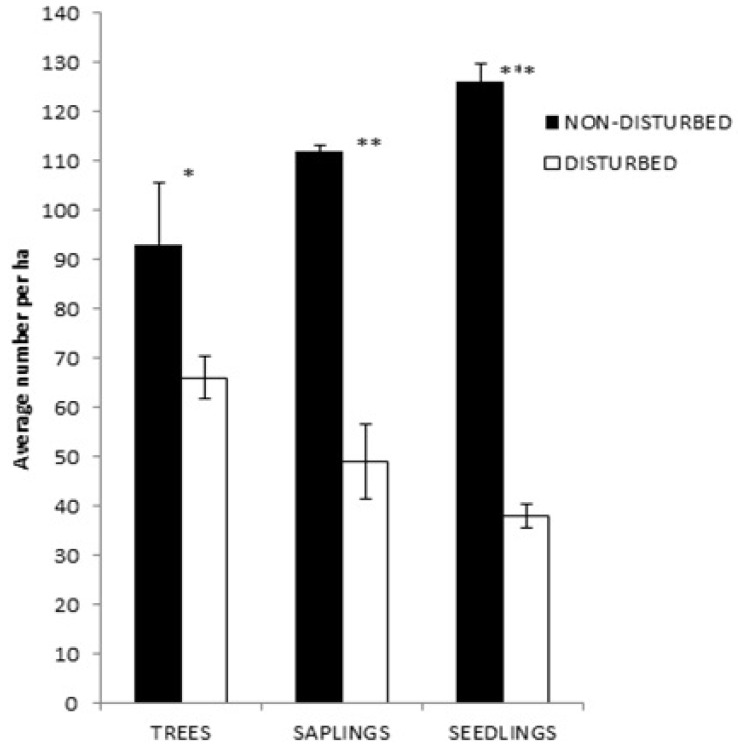
Average ±SE number of stems of mature trees, saplings, and seedlings per ha across all tree species plotted along the disturbed and non-disturbed sites. Asterisks above bars signpost significant differences within the growth stages across the disturbed and non-disturbed sites according to Tukey’s Post-Hoc tests (* = *p* < 0.05; ** = *p* < 0.01; *** = *p* < 0.001).

We found a vigorous natural regeneration at the non-disturbed site, where saplings and seedlings together represented more than 72% of the site population, while the disturbed site was dominated by mature trees with less than 15% saplings and seedlings (Figure 4). The growth form distributions (mature tree, sapling, and seedling) were significantly different within and between the disturbed and non-disturbed sites, with an inverse distribution pattern (*F_2,48_* = 116.4, *p* = 0.034 and *F_2,48_* = 163.2, *p* = 0.021, respectively; Figure 4).

### 2.4. High Density and Crown Width of Balanites aegyptiaca in the Non-Disturbed Site

The density of *B. aegyptiaca* in the disturbed site was less than half that of the non-disturbed site (Table 1) while its tree crowns were twice as wide in the non-disturbed than in the disturbed sites. The basal area contribution (%) for *B. aegyptiaca* was significantly more aggregated in the disturbed site and more regularly spaced in the non-disturbed site (Blackman index, Table 1).

**Figure 5:**
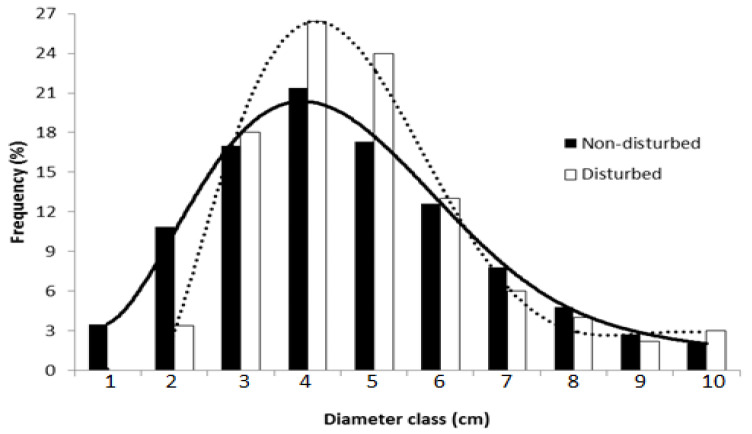
Weibull distribution for the tree diameter at breast height (DBH) of *B. aegyptiaca* in the non-disturbed and disturbed sites at the Dinder Biosphere Reserve, Sudan, along increasing DBH categories. Classes: 1 = 6–10 cm, 2 = 11–15 cm, 3 = 16–20 cm, 4 = 21–25 cm, 5 = 26–30 cm, 6 = 31–35 cm, 7 = 36–40 cm, 8 = 41–45 cm, 9 = 46–50 cm, 10 = 51–55 cm, and *n* = 545.

**Figure 6:**
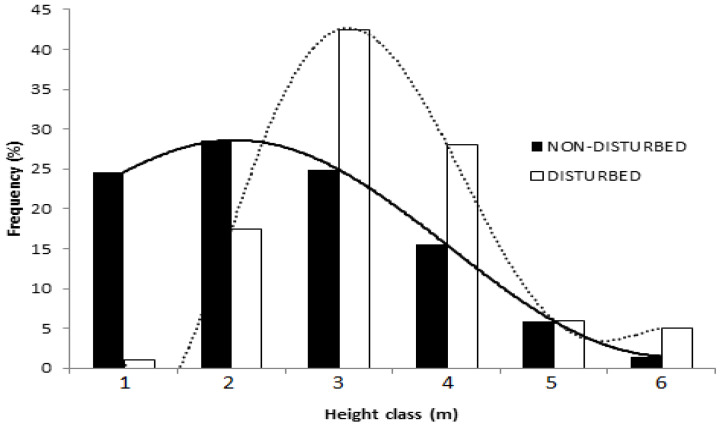
Weibull distribution for the tree height of *B. aegyptiaca* in the non-disturbed and disturbed sites at the Dinder Biosphere Reserve, Sudan, along increasing height categories. Classes: 1 = 3–5 m, 2 = 6–8 m, 3 = 9–11 m, 4 = 12–14 m, 5 = 15–17 m, 6 = 18–20 m, and *n* = 545.

**Figure 7:**
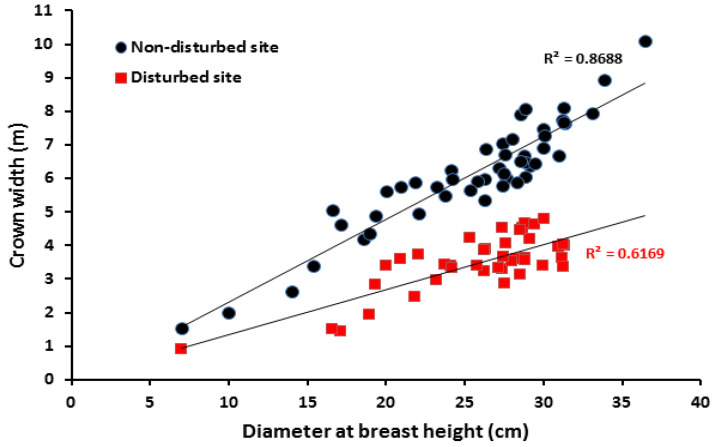
Correlation between crown width and diameter at breast height for *B. aegyptiaca* in the disturbed and non-disturbed sites at the Dinder Biosphere Reserve, *n* = 545.

The diameter at breast height (DBH) of *B. aegyptiaca* in the non-disturbed site exhibited a bell-shaped normal distribution while it resembled an inverse J-shape, positive asymmetrical Weibull distribution in the disturbed site (W = 0.853; Figure 5). The distribution of height classes displayed a different trend, with juvenile trees dominating in the non-disturbed site and mature trees in the disturbed one (W = 0.845; Figure 6). Further, the correlation between crown width and DBH showed a strong positive relationship with *R*^2^ = 0.6 for both the disturbed and non-disturbed sites (Figure 7). At the disturbed sites, the slope of crown width vs. DBH was lower, indicating that the site was dominated by the medium-sized trees.

## 3. Discussion

### 3.1. Low Tree Species Diversity and Density in Disturbed Sites

Our findings revealed that the disturbed site has lower species richness, diversity, and evenness compared to the non-disturbed site, which is consistent with [1,12]. Only a few tree species dominated the disturbed sites, which might be more tolerant to livestock browsing. However, as tree diversity is an essential component for a healthy forest system [45,46], this limited number of dominant tree species raises a critical concern. Researchers [47] concluded that 76% of perennial plants in southern Jeddah, Saudi Arabia, disappeared due to ecological and anthropogenic disturbances, while [48] reported that tree species diversity and richness of Slovenian forests were reduced from 272 to 243 species because of deer browsing and livestock grazing. In contrast, [49] documented that low and intermediate disturbances increased the tree species richness, diversity, and functional richness of the boreal forests in Ontario. Our low species richness and diversity in the disturbed site may be attributed to the intensive livestock browsing and illegal harvesting in the area. Accordingly, controlling livestock grazing and human trespassing in the Dinder Biosphere Reserve is crucial for minimizing this pressure and paving the way for tree recovery. We recommend that the introduction of community forestry in the degraded areas around the villages can contribute efficiently to controlling harvesting activities in reserves. However, we claim that awareness-raising programs are also urgently needed to reduce the current and future damage to natural regeneration in the reserve.

The inverse pattern of *Anogeissus leiocarpus* showing more seedlings and saplings in the disturbed sites and more adults in the non-disturbed site might be due to the intensive illegal harvesting of adult trees by the local community, as this species seems in high demand across eastern Africa [50,51,52,53]. Further, the high tannin content in its seedlings and saplings may reduce its palatability for livestock, and, hence, might have minimized the browsing impact [54].

### 3.2. High Importance Value Index (IVI) and Dominance in Disturbed Sites

Our results elucidate that *Acacia leata, A. mellifera* (Vahl) Benth, *A. polyacantha, A. senegal,* and *A. seyal var fistula* were frequent at the disturbed site. These tree species are characterized by a high growth rate and the ability to quickly encroach degraded and open spaced rangeland [55,56]. Hence, our disturbed areas might already show signs of bush encroachment through these species due to the intensive anthropogenic use. Accordingly, high densities of these *Acacia* species, combined with the ongoing illegal felling and livestock browsing, may compromise the recruitment of *B. aegyptiaca* seedlings and saplings in the disturbed site and eliminate its population, as was found for fruit and nut tree species [26,57,58]. The overutilization by livestock, bush encroachment, and over-harvesting have also led to the degradation of useful tree species and a biodiversity decline in Ethiopia, Tanzania, and Botswana, respectively [56,57,58]. Our high value of IVI for *Acacia seyal* trees in disturbed sites, with a huge gap to the nearest tree species, is an indicator of the unbalanced ecosystem and needs urgent action to protect the vulnerable tree species in the area.

An unbalanced ecosystem generally appears as a result of biotic and abiotic disturbances or both, within or across different species [29,47,59]. For the disturbed sites in the DBR, both types were observed; the aggregated distribution patterns of *B. aegyptiaca* illustrated that seedlings and saplings were located in patches. In addition, the across-species trend was shown by the low tree species richness in the disturbed compared to the non-disturbed sites.

Furthermore, the tree stand density is linearly proportional to the basal area, a key parameter of relative occurrence (relative dominance) [60]. Our results highlight that tree species such as *B. aegyptiaca*, *T. brownii*, and *T. indica* have been over-utilized and their populations severely disturbed. These results are in line with [34] and [60,61], who concluded that illegal harvesting and livestock browsing affected the stand density and the IVI in the natural forests in Sudan.

### 3.3. Natural Regeneration and Growth form Distribution Limited in Disturbed Sites

We found that more than 85% of the tree species in the disturbed site have a poor regeneration status, and some species such as *T. indica* were only found in their mature stage. In contrast, seedlings and saplings of *B. aegyptiaca, Combretum hartmannianum, A. seyal, Anogeissus leiocarpus,* and *Lannea fruticosa* represented 72% of the tree population at the non-disturbed site. With the assumption that all tree species in DBR are native [14,62], disturbances resulting from environmental factors should affect them equally. Wild animals are present in the non-disturbed site and the buffer zone of the DBR [17,63,64], where we did not find any reduced tree recruitment. Therefore, the reduction in natural regeneration and crown width we found in our disturbed site likely results from intensive browsing by livestock and a reduction of seed production as branches are cut and edible fruits are collected by the local communities. Our findings are in line with previous studies in Kenya and Sudan [2,60,61], where felling mainly affected mature trees while new recruitment was hampered by over-browsing by livestock.

Intensive browsing by livestock influences the recruitment of the tree seedlings and saplings and disturbs the population dynamics [2,19,65]. The density of seedlings of *Quercus ithaburensis* Decne and *Quercus agrifolia* Ne’e in Israel and north California, respectively, declined to <50% of the initial population due to intensive browsing and overgrazing of livestock [66,67] and a 50% tree species decline was observed in the Galician oak forest of Northwest Spain [68]. In our study, the low percentage of seedlings and saplings in the disturbed sites (15%) indicates that the recruitment of seedlings and saplings to adult trees was severely interrupted. Likewise, illegal harvesting through the cutting of branches, debarking of the stem, and the removal of entire trees eliminate the vulnerable tree species and reduce the species diversity in the highly disturbed stands [1,12,60], which we could also see for *B. aegyptiaca* tree species. Therefore, we highlight that all areas with new regeneration of *B. aegyptiaca* and other vulnerable tree species in the transition zone of the DBR must be strongly protected to allow natural recovery. In our study, we did not include the effect of forest fire and drought, which might also reduce the success of natural regeneration and damage tree seedlings and saplings [20,69,70] as there was only limited information on these factors. However, we can show that already, human and livestock activities alone leave a significant negative effect on recruitment and persistence of most tree species in the DBR, highlighting the fragility of this ecosystem.

### 3.4. High Density and Crown Width of Balanites aegyptiaca in the Non-Disturbed Sites

We found the high density and crown width but the low basal area contribution of *B. aegyptiaca* in the non-disturbed sites. This situation might result from seasonal foraging patterns as, during the dry season, pastoralists and livestock keepers debranch mature and taller trees for livestock to browse [1,71,72]. During the wet season, livestock utilize and depend on perennial and herbaceous plants that are generally available in and around the villages [41,47,73]. A healthy population structure is usually characterized by higher seedling and sapling (young generation) proportions compared to mature trees [2,19,60]. Our results of higher seedling and sapling densities in the non-disturbed sites agree with these conclusions. Moreover, the lower slope of the correlation between the crown width and DBH of *B. aegyptiaca* in the disturbed site demonstrates that even healthy trees might be under high competition in disturbed sites and cannot fully expand for effective photosynthesis.

The variation in class frequency distribution across disturbance sites may result from the massive medicinal uses of *B. aegyptiaca* by local people as they prune the foliage, debark the stem and dig for tree roots [31,34,74]. Intensive pruning and bark removal will reduce the quantity of foliage and interrupts tree growth [1]. Although minor disturbance can enhance the biological diversity in natural forests [75], extensive use may accelerate forest degradation [71,76]. While our study is only a snapshot in time, i.e., covering only one year of assessment, it provides information that can be used to initiate comprehensive monitoring and awareness-raising programs about the current degraded situation and help managing the reserve on a sustainable basis, particularly *B. aegyptiaca* stands.

Other factors that might cause a fluctuation in natural regeneration are the type of browsers that dominate the site [21,77]. Goats are characterized by an extensive browsing habit with the preference for woody plants and broadleaved tree species [77,78,79], while cattle and camels prefer grazing on herbaceous plants and browsing the crown of mature trees, respectively [19,21,80]. Although grasses might protect small tree seedlings from browsing livestock [81,82], in the DBR, only little grass cover remained during the dry season, and grasses were severely overgrazed by livestock [14,73,83]. Researchers [78] have documented that more than 85% of *Pinus sylvestris* L saplings in Mediterranean mountains were browsed more than once per season by goats, and more than 30% of the apical shoot had been consumed after establishment. Such browsing activity can eliminate the species in its critical stage of development and hinder the tree population dynamic, particularly for vulnerable species such as *B. aegyptiaca*.

## 4. Materials and Methods

### 4.1. Study Area

**Figure 8:**
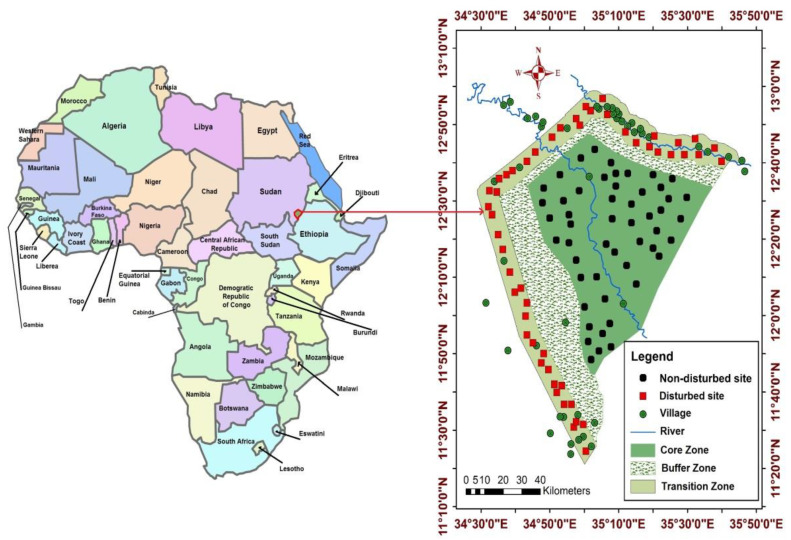
Map of the Dinder Biosphere Reserve, Sudan, including the sampling locations at the disturbed and non-disturbed sites during our assessment of tree species composition and growth characteristics of *B. aegyptiaca* over one year from April 2019 to April 2020.

The study was conducted in the Dinder Biosphere Reserve (DBR), Sudan, located at 12°26′ N, 12°42′ N, 34°48′ E and 35°02′ E (Figure 8), with a total area of 10,291 km^2^ [73]. The average monthly minimum and maximum temperatures were 18 °C and 30 °C, respectively, while the average annual rainfall was 775 mm [64,84]. The DBR consists of three zones, which are the transition, buffer, and core zone [17,18] (Figure 8). The transition zone is under high human pressure, encompassing more than 20 villages (disturbed), while the core zone is fully protected (non-disturbed) [73]. The disturbed site is under high anthropogenic pressure by frequent illegal harvesting, livestock grazing and agriculture [18,73] while at the non-disturbed site, timber harvesting, pastoral and agricultural as well as charcoal production are prohibited [14,73].

### 4.2. Data Collection

We randomly laid out 100 rectangular sample plots of 25 m × 40 m (1000 m^2^) across the study area, 50 each in the disturbed and non-disturbed site, respectively, from April 2019 to April 2020. Within each sample plot, we identified all tree species and grouped them into adult trees with a stem diameter at breast height (diameter at 1.3 m above the ground) of DBH > 7 cm [60], saplings (core diameter < 7 cm and >3 cm; [10]) and seedlings (core diameter < 3 cm) and a height of H < 1 m [85,86]. For all adult trees, we measured the DBH, total tree height (H), crown width (CW) and crown height (CH). Calipers (65 cm) were used for measuring the diameter at breast height (DBH) for the small trees and diameter tapes (5 m) for the larger ones. We used a Suunto clinometer for measuring H and a Spiegel Relaskope for the measurements of CH as recommended by [86,87,88]. The CW was measured by using a tape measure (50 m) in eight directions from the main tree stem (every 45 degree) to the vertically projected edge of the crown [88]. All assessed tree species in this study were identified by a botanist and the literature [28,89].

### 4.3. Data Analysis

For each sample plot, we calculated the means of the tree size parameters (diameter at breast height, height, and crown width) as recommended by [1,12]. We also computed tree basal area (m^2^), tree density (number of trees ha^−1^), and importance value index (IVI) [1,12,60,61], (Table 2). This index results from the integration of relative frequency, relative abundance, and relative dominance, which are all expressed in percentages [1]. The values of IVI vary from 0 to 300, with 0 indicating no importance while values close to 300 show high importance of the species within the tree species community [12,90].

The species richness (*S*) was calculated as the number of species recorded in each site [1]. Moreover, to measure the stand diversity, Simpson’s reciprocal Index of Diversity (*I_D_*) and Simpson’s evenness (*Eq*) were calculated by applying the following equations [1,12]:

(1) $$\mathrm{Simpson}’s\;\mathrm{reciprocal}\;\mathrm{Index}\;(I_{D})\;=\;\;\frac{1}{\sum_{i=1}^{sp}\frac{n_{i}\left(n_{i}\;-\;1\right)}{n\left(n-1\right)}},$$

(2) $$\mathrm{Simpson}’s\;\mathrm{evenness}\;(Eq)\;=\;\;\frac{I_{D}}{I_{max}}\;\mathrm{with}\;I_{max}=S,$$

*n_i_* is the number of trees of species *i*, *n* is the overall number of trees cruised in the plot, and *sp* is the number of species found in the considered plot. *I_max_* represents the maximum value of the Simpson’s diversity index and *S* is the species richness.

The mean and coefficient of variation for all calculated parameters were computed for all sample plots and species by using Minitab 17. One-way ANOVA in JAMOVI (version 1.1.7) was performed to compare mean numbers of trees, saplings, and seedlings in the non-disturbed and disturbed sites. A paired sampled *T*-test was accomplished using JAMOVI to compare the studied dendrometric parameters between the sites. The same tests also applied for the comparison between diversity indices in the two studied sites as recommended by [1,12]. Further, we ran a hierarchical cluster analysis (HCA) in Past (version 3.6) to group the values of IVI, and the three resulted groups for both the disturbed and non-disturbed sites were projected in the principal component analysis (PCA).

Weibull distribution was used to distinguish between size class distributions of *B. aegyptiaca* in the disturbed and non-disturbed sites [1,12] in Minitab 17.

## 5. Conclusions

Our study findings elucidated that the disturbed sites in the Dinder Biosphere Reserve have low species richness, diversity and natural regeneration compared to the non-disturbed sites. Moreover, vulnerable species such as *B. aegyptiaca* are severely impacted by anthropogenic disturbances, and their natural regeneration might be at risk. The results also revealed that *A. seyal* was the most dominant tree species in the disturbed site, which may show its bush encroachment properties [55,56]. We also demonstrated that more than 85% of the tree species in the disturbed sites have poor natural regeneration, which may mainly result from the intensive livestock grazing and browsing. We showed that species such as *Tamarindus indica*, *Adansonia digitata*, *Terminalia brownii*, and *Piliostigma reticulatum* have the lowest importance value index, and even, some of them are found only in the mature stage. Such species need urgent protection and conservation measures to recover and restore naturally before they collapse and disappear.

Furthermore, we found a low tree diversity and natural regeneration, particularly in areas that were exposed to intensive goats browsing (see also Mohammed et al. 2021, in review). Goats generally browse a diversified range of tree species at different heights from close to ground level up to the height of 2.10 m [21], which makes goats the most harmful livestock to natural regeneration.

We claim that the current policy of managing the transition zone (disturbed site) of the Dinder Biosphere Reserve must be revised, and more restrictions should be endorsed to eliminate this intensive illegal harvesting. We further recommended a comprehensive monitoring program in the transition zone (disturbed site) to control the browsing of livestock within the reserve and promote the natural regeneration of *B. aegyptiaca* and other vulnerable tree species.

## Figures and Tables

**Table 1:** Mean (±SE) of the dendrometric parameters and Blackman index of *Balanites aegyptiaca* trees in the non-disturbed and disturbed sites at the Dinder Biosphere Reserve collected over one year. DBH = diameter at breast height (measured at 1.3 m above ground level). SE = standard error, *T* = paired sample *t* test, *p* = probability value.

Parameter	Non-Disturbed Site	Disturbed Site	*T*	*p*
	Mean (±SE)	Mean (±SE)		
Tree DBH (cm)	32.8 ± 0.7	27.5 ± 0.4	2.1	0.038
Tree height (m)	12.5 ± 0.2	9.6 ± 0.1	3.6	0.001
Tree crown width (m)	7.7 ± 0.2	3.3 ± 0.1	3.2	0.002
Tree basal area (m^2^ ha^−1^)	0.07 ± 0.03	0.06 ± 0.02	2.1	0.034
Tree volume (m^3^)	0.39 ± 0.02	0.27 ± 0.10	2.9	0.004
Tree density (stem ha^−1^)	18.7 ± 0.4	8.9 ± 1.2	2.6	0.018
Basal area contribution (%)	9.5 ± 0.3	23.1 ± 1.3	30.8	<0.001
Blackman index	0.52 ± 0.30	4.02 ± 0.40	39.3	<0.001

**Table 2:** Utilized equations for the calculation of dendrometric and structural parameters of the tree species collected in the Dinder Biosphere Reserve, Sudan, over a period of one year in sites of different anthropogenic pressure. *DBH_i_* is the diameter at breast height of the *i*th tree (cm), *n* represents the total number of trees in the sample, and *s* is the area sampled. $\sigma_{N}^{2}$ and $\mu_{N}$ represent the variance and mean, respectively, of the *B. aegyptiaca* tree density of the site. Density is the number of stems per sampled area. *G_B_* is the basal area of *B. aegyptiaca* trees while *G* is the basal area for all tree species encountered per site (stand basal area).

Parameter	Equation	Reference
Tree basal area (g)	g = (π ÷ 4) × *DBH_i_*^2^	[61]
Stand basal area (G)	G = (*n* ÷ 4 *s*) × ∑*DBH_i_*^2^	[12]
Stand density (N)	N = *n* ÷ *s*	[1]
Species abundance (A)	A = Total number of trees for a species ÷ Area sampled	[61]
Relative abundance (RA)	RA = (Abundance of a species ÷ Total abundance for all) × 100	[12]
Species dominance (D)	D = Total basal area of a species ÷ area sampled	[61]
Relative dominance (RD)	RD = (Coverage of a species ÷ Total coverage for all) × 100	[12]
Species frequency (F)	F = Occurrence or absence of species in a sample plot	[60]
Relative frequency (RF)	RF = (Frequency of a species ÷ Total frequency for all) × 100	[61]
Importance Value Index	IVI = RA + RD + RF	[12]
Blackman Index (IB)	IB = $\sigma_{N}^{2}$ ÷ $\mu_{N}$	[1]
Basal area contribution	Cs = (*G_B_ ÷ G*) × 100	[1]

## Appendix A

**Table A1:** Total number, relative abundance, dominance, frequency and IVI of the tree species counted and measured in the disturbed site in the Dinder Biosphere Reserve.

Species	*N*	Relative Abundance (%)	Relative Dominance (%)	Relative Frequency (%)	IVI
*Acacia leata*	26	1.0	0.5	3.4	4.9
*Acacia mellifera*	56	2.2	1.0	3.9	7.1
*Acacia polyacantha*	35	1.4	0.8	2.0	4.1
*Acacia senegal*	144	7.8	6.2	7.8	21.8
*Acacia seyal var fistula*	65	2.6	1.7	3.9	8.2
*Acacia seyal var seyal*	514	27.8	37.1	14.5	79.4
*Adansonia digitata*	10	0.4	1.1	2.0	3.4
*Anogeissus leiocarpus*	97	3.8	3.9	2.9	10.7
*Balanites aegyptiaca*	187	10.1	13.6	12.7	36.4
*Combretum hartmannianum*	226	12.2	4.4	11.5	38.1
*Dichrostachys cinerea*	41	1.6	0.7	3.4	5.8
*Lannea fruticosa*	52	2.1	0.9	2.4	5.4
*Lonchocarpus laxiflorus*	171	9.3	3.5	6.6	19.4
*Sterculia setigera*	40	1.6	1.7	2.0	5.2
*Tamarindus indica*	12	0.6	0.8	2.0	3.4
*Terminalia brownii*	25	1.0	0.7	1.5	3.2
*Ziziphus spina-christi*	142	7.7	6.4	10.8	24.9

*N* = Total number of trees per site; IVI = Importance value index for each tree species per site.

**Table A2:** Total number, relative abundance, dominance, frequency and IVI of the tree species counted and measured in the non-disturbed site in the Dinder Biosphere Reserve.

Species	*N*	Relative Abundance (%)	Relative Dominance (%)	Relative Frequency (%)	IVI
*Acacia polyacantha*	54	2.2	0.8	2.0	5.0
*Acacia senegal*	161	5.5	4.1	3.3	12.9
*Acacia seyal var fistula*	27	1.1	0.4	1.1	2.6
*Acacia seyal var seyal*	319	10.9	9.1	8.4	28.4
*Anogeissus leiocarpus*	182	7.4	8.8	5.1	21.3
*Balanites aegyptiaca*	358	16.7	17.8	13.5	48.0
*Boscia senegalensis*	31	1.3	0.5	3.6	5.3
*Boswellia papyrifera*	96	2.5	3.5	3.0	9.0
*Combretum aculeatum*	30	1.2	0.5	3.6	5.2
*Combretum ghasalense*	67	2.7	2.7	2.6	7.9
*Combretum glutinosum*	87	3.5	3.5	4.0	11.0
*Combretum hartmannianum*	321	11.0	14.8	6.4	36.2
*Commiphora africana*	32	1.3	1.9	2.3	5.5
*Dalbergia melanoxylon*	45	1.8	1.2	2.6	5.5
*Dichrostachys cinerea*	31	1.3	0.5	4.0	5.8
*Diospyros mespiliformis*	44	1.8	1.6	2.0	5.4
*Gardenia lutea*	36	1.5	0.6	3.6	5.7
*Hyphaena thebiaca*	49	2.0	2.9	2.6	7.4
*Lannea fruticosa*	184	4.5	4.4	5.6	14.5
*Lannea nigritana*	91	3.7	3.4	3.7	10.8
*Lannea schimperi*	36	1.5	1.9	1.7	5.1
*Maerua angolensis*	30	1.5	0.6	2.8	5.3
*Piliostigma reticulatum*	20	0.8	0.6	1.1	2.6
*Pseudocedreca kotschyi*	58	2.4	2.7	3.4	8.5
*Pterocarpus lucens*	50	2.0	3.1	2.0	7.1
*Sclerocarya birrea*	48	2.0	2.6	2.3	6.8
*Sterculia setigera*	84	3.4	5.0	4.0	12.3
*Stereospermum kunthianum*	16	0.7	0.9	1.1	2.6
*Strychnos innocua*	28	1.1	0.7	3.6	5.4
*Tamarindus indica*	19	0.8	1.0	0.9	2.6
*Terminalia brownii*	14	0.6	0.7	0.6	1.9
*Terminalia macroptera*	52	2.1	3.1	2.3	7.5
*Xeromphis nilotica*	29	1.5	0.4	3.7	5.6
*Ximenia americana*	41	1.7	0.9	2.8	5.3
*Ziziphus spina-christi*	154	5.3	3.0	5.1	13.4

*N* = Total number of trees per site; IVI = Importance value index for each tree species per site.

**Table A3:** Total number, relative abundance, dominance, frequency and the Importance Value Index calculated for each species per site (IVI) for the seven most frequently encountered tree species in non-disturbed and disturbed sites at the Dinder Biosphere Reserve, Sudan, during the assessment in February–April 2020. Non = Non-disturbed site; Dis = Disturbed site. Calculations were carried out for mature trees, seedlings and saplings of each species combined.

Species	*N*		Relative Abundance (%)		Relative Dominance (%)		Relative Frequency (%)		IVI	
	Non	Dis	Non	Dis	Non	Dis	Non	Dis	Non	Dis
*Acacia senegal*	161	144	5.5	7.8	4.1	6.2	3.3	7.8	12.9	21.8
*Acacia seyal var seyal*	319	514	10.9	27.8	9.1	37.1	8.4	14.5	28.4	79.4
*Anogeissus leiocarpus*	182	97	7.4	3.8	8.8	3.9	5.1	2.9	21.3	10.7
*Balanites aegyptiaca*	358	187	16.7	10.1	17.8	13.6	13.5	12.7	48.0	36.4
*Combretum hartmannianum*	321	226	11.0	15.3	14.8	17.4	6.4	12.6	36.2	45.3
*Lannea fruticosa*	184	52	4.5	2.1	4.4	0.9	5.6	2.4	14.5	5.4
*Ziziphus spina-christi*	154	142	5.3	7.7	3.0	6.4	5.1	10.8	13.4	24.9

*N* = Total number of trees per site; IVI = Importance value index for each tree species per site.
